# PDE9A deficiency does not prevent chronic‐hypoxic pulmonary hypertension in mice

**DOI:** 10.14814/phy2.15057

**Published:** 2021-09-27

**Authors:** Todd M. Kolb, Laura Johnston, Mahendra Damarla, David A. Kass, Paul M. Hassoun

**Affiliations:** ^1^ Division of Pulmonary and Critical Care Medicine Pulmonary Johns Hopkins University Baltimore Maryland USA; ^2^ Division of Cardiology Department of Medicine Johns Hopkins University Baltimore Maryland USA

**Keywords:** chronic hypoxia, mouse model, phosphodiesterase 9A, pulmonary hypertension

## Abstract

Inhibition of cyclic guanosine monophosphate (cGMP)‐specific phosphodiesterases (PDEs) is a cornerstone of pulmonary arterial hypertension (PAH)‐specific therapy. PDE9A, expressed in the heart and lung tissue, has the highest affinity for cGMP of all known PDEs. PDE9A deficiency protects mice against chronic left ventricular (LV) pressure overload via increased natriuretic peptide (NP)‐dependent cGMP signaling. Chronic‐hypoxic pulmonary hypertension (CH‐PH) is a model of chronic right ventricular (RV) pressure overload, and previous studies have demonstrated a protective role for NPs in the murine model. Therefore, we hypothesized that PDE9A deficiency would promote NP‐dependent cGMP signaling and prevent RV remodeling in the CH‐PH model, analogous to findings in the LV. We exposed wild‐type and PDE9A‐deficient (*Pde9a* ^−/−^) C57BL/6 mice to CH‐PH for 3 weeks. We measured RV pressure, hypertrophy, and levels of lung and RV cGMP, PDE9A, PDE5A, and phosphorylation of the protein kinase G substrate VASP (vasodilatory‐stimulated phosphoprotein) after CH‐PH. In wild‐type mice, CH‐PH was associated with increased circulating ANP and lung PDE5A, but no increase in cGMP, PDE9A, or VASP phosphorylation. Downstream effectors of cGMP were not increased in *Pde9a* ^−/−^ mice exposed to CH‐PH compared with *Pde9a* ^+/+^ littermates, and CH‐PH induced increases in RV pressure and hypertrophy were not attenuated in knockout mice. Taken together, these findings argue against a prominent role for PDE9A in the murine CH‐PH model.

## Graphical abstract

Phosphodiesterase (PDE) 9A deficiency protects mice from chronic left ventricular pressure overload by augmenting NP‐dependent signaling. Chronic‐hypoxic PH is a model of chronic right ventricular pressure overload, and NP overexpression has previously been shown to be protective. PDE 9A deficient mice did not have increased NP‐dependent signaling and were not protected from chronic‐hypoxic PH.

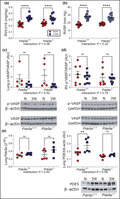

## 1 INTRODUCTION

Inhibiting specific phosphodiesterase (PDE) enzymes to augment cyclic guanosine monophosphate (cGMP)‐dependent signaling is a cornerstone of therapy in pulmonary arterial hypertension (PAH) (Archer & Michelakis, 2009). The intracellular second messenger cGMP plays a critical role in regulating endothelial, vascular smooth muscle, and cardiac myocyte function (Tsai & Kass, 2009). Activation of cGMP‐dependent signaling generally promotes vaso‐relaxation and myocardial contractility, while preventing hypertrophy and proliferation. These beneficial effects are primarily mediated via cGMP‐dependent protein kinase (PKG) (Hofmann et al., 2009), while concurrent activation of PDE isoforms can hydrolyze cGMP and effectively downregulate the signal (Omori & Kotera, 2007). In PAH, PDE5 inhibitors (sildenafil, tadalafil, vardenafil) reduce pulmonary vascular resistance and increase exercise capacity (Galie et al., 2009, 2005; Jing et al., 2011) but well‐described counterregulatory mechanisms (Black et al., 2001; Giaid & Saleh, 1995; Murray et al., 2002; Xu et al., 2004) may mitigate benefits for some patients.

PDE‐9A is expressed in heart and lung and has the highest affinity for cGMP of all known PDE isoforms (Soderling et al., 1998). Myocardial PDE9A is upregulated in left heart failure, and PDE9A‐deficient mice had less myocyte hypertrophy and better myocardial function than PDE9A‐expressing mice in a model of chronic left ventricular (LV) pressure overload (Lee et al., 2015). The beneficial effects of PDE9A deficiency in this model were mediated through augmentation of natriuretic peptide (NP)‐dependent cGMP signaling. Atrial natriuretic peptide (ANP) overexpression is protective in the mouse model of chronic hypoxic pulmonary hypertension (CH‐PH) (Klinger et al., 1993), while ANP knockout mice develop more severe CH‐PH (Chen et al., 2006; Klinger et al., 1999; Sun et al., 2000). The role of PDE9A in murine CH‐PH is unknown. We hypothesized that PDE9A deficiency would promote NP‐dependent cGMP signaling and attenuate CH‐PH.

## 2 METHODS

### 2.1 Mice and CH‐PH Model

All protocols were approved by the Johns Hopkins Animal Care and Use Committee. Wild‐type C57BL/6J mice (Black et al., 2001; Giaid & Saleh, 1995; Murray et al., 2002) were obtained from Jackson Laboratories. *Pde9a* knockout mice (*Pde9a* ^−/−^) were developed on the C57BL/6J background by Pfizer by replacing exon 12 of the *Pde9a* catalytic domain (expressed by all splice variants) with a *lacZ*‐neomycin cassette (Figure S1), as previously reported in Lee et al. (2015). In experiments using knockout mice, age‐matched *Pde9a* ^+/+^ littermates were used as controls, and males and females were included.

To generate CH‐PH, we housed mice in a ventilated plexiglass chamber under conditions of normobaric hypoxia (10% O_2,_ balance nitrogen; Pro‐Ox 110; Biospherix) for 3 weeks. Excess CO_2_ was scavenged with soda lime. The chamber was opened twice weekly for cage changes. Normoxic control mice were housed in the same room, outside the chamber. Food and water were available ad libitum. Mice were weighed prior to sacrifice and exsanguinated under anesthesia with pentobarbital (60 mg/kg IP). Blood was collected and serum separated by centrifugation. The lungs and heart were removed, and the RV free wall was dissected from the LV and septum (LV +S). We quantified right ventricular hypertrophy (RVH) using the ratio of RV mass to LV +S mass (Fulton index) and body weight. Serum and tissues were snap‐frozen in liquid N_2_ for subsequent biochemical and molecular analyses.

### 2.2 Hemodynamic measurements

In some experiments, we obtained RV pressure tracings after 3 weeks of CH‐PH using a high‐fidelity pressure sensor tipped catheter (Transonic Systems, Inc.). During these measurements, anesthesia was maintained with pentobarbital, and body temperature was continuously maintained at 37℃ (±0.4°) using a proportional‐integral‐derivative temperature control unit. Mice were intubated via tracheostomy and mechanically ventilated (room air) using a tidal volume of 10 cc/kg and a respiratory rate of 160 breaths/min. After confirming adequate anesthesia (paw pinch), the diaphragm was exposed using a horizontal incision just below the xiphoid. Following diaphragmotomy, the pericardium was removed and the RV was punctured with a 25‐gauge needle. A 1.2 F catheter was inserted into the RV, and continuous pressure tracings were recorded during a brief (5 s) pause in mechanical ventilation. Pressure tracings were analyzed post‐ hoc using LabChart Pro 7 software (ADInstruments).

### 2.3 Serum ANP measurement

Circulating ANP levels were measured from serum using a commercially available ELISA (Sigma‐Aldrich), according to the manufacturers’ protocol. The company reports an assay sensitivity of 1.02 pg/mL.

### 2.4 Tissue cGMP measurement

Frozen tissue samples were homogenized in 0.1 N HCl, and cGMP levels were measured using a commercially available competitive ELISA (Enzo Life Sciences). The company reports an assay sensitivity of 0.420 pmol/mL, with <0.001% reactivity with cAMP.

### 2.5 Immunoblotting

Frozen tissue samples were ground under liquid nitrogen, then homogenized by mechanical disruption using the Bullet Blender Tissue Homogenizer (Next Advance, Inc.) in RIPA buffer (Cell Signaling Technology) supplemented with protease (cOmplete Mini) and phosphatase (PhosSTOP) inhibitor cocktails (Roche). Homogenates were cleared by centrifugation at 12,000 *g* for 15 min. Following brief sonication, the colorimetric bicinchoninic acid (BCA) assay (Thermo Fisher Scientific) was used to quantify protein samples for equivalent gel loading. Electrophoresis and immunoblot analysis of tissue homogenates was performed with standard methods and commercially available antibodies (Cell Signaling Technology) for PDE5A (#2395; 1:1000), VASP (#3112; 1:1000), phospho‐VASP (ser 239, #3114; 1:1000), β‐actin (#5125; 1:5000), GAPDH (#8884; 1:5000), and hsp90 (#4874; 1:1000). We quantified band intensity using ImageJ software (Schneider et al., 2012) and normalized band intensity to a reference protein (β‐actin, GAPDH, or hsp90) or the parent kinase (for VASP phosphorylation).

### 2.6 PCR

For quantitative PCR, we isolated total RNA from frozen tissues with Trizol (Thermo Fisher Scientific) and purified it with a commercially available kit (QIAgen). Subsequently prepared cDNA (GE First‐Strand cDNA Synthesis Kit) was used as the template for quantitative PCR using the BIORAD iQ5 thermal cycler (BIORAD). Primer sets for mouse *Pde9a* (#PPM25629A), *Pde5a* (#PPM33467A), *Nppa* (#PPM04489A), *Npr3* (#PPM29599A), and *Gapdh* (#PPM02946E) were purchased from QIAgen. Background subtracted amplification data were analyzed using open‐source software (Ruijter et al., 2009) to estimate Ct values and amplification efficiency. Target gene expression was normalized to a reference gene using the comparative Ct method (Schmittgen & Livak, 2008). For genotyping of *Pde9a* knockout mice, we isolated genomic DNA from mouse tails using a commercially available kit (QIAgen) and performed PCR using primers designed between exons 11 and 13, including neomycin, as shown in Figure S1a: GS1 (5′‐CACAGATGATGTACAGTATGGTCTGG‐3′), GS2 (5′‐TGCAGTCATCAGGACCAAGATGTCC‐3′), and Neo (5′‐GACGAGTTCTTCTGAGGGGATCGATC‐3′).

### 2.7 Statistical analysis

We performed all statistical analyses using GraphPad Prism 8 software (GraphPad Software). For comparison of means between two groups, we used Student's *t*‐test. For comparison of means in experiments using knockout mice, we performed two‐way ANOVA with genotype (*Pde9a* ^−/−^ vs. *Pde9a* ^+/+^) and exposure (normoxia vs CH‐PH) as the variables. Post hoc comparisons of means within each genotype were completed using Sidak's multiple comparison test. In all analyses, we considered a *p* < 0.05 statistically significant.

## 3 RESULTS

### 3.1 CH‐PH is associated with increased circulating ANP

**FIGURE 1:**
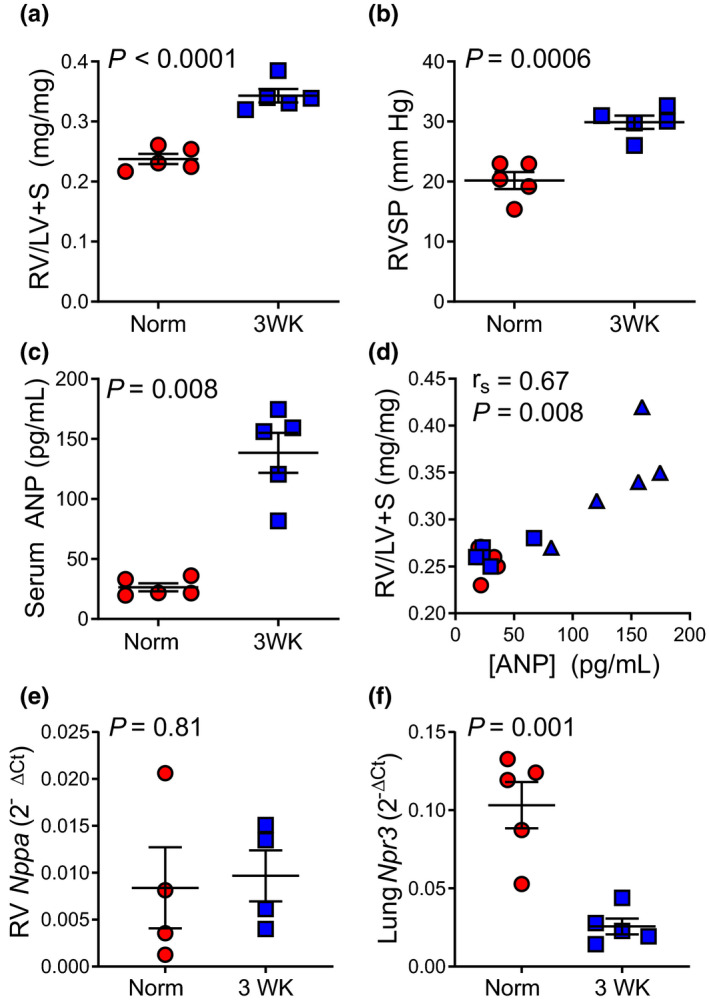
Mechanism of ANP increase in CH‐PH model. After 3 weeks of exposure to chronic hypoxia, RV mass (relative to LV +S = Fulton Index; (a) and RV systolic pressure (RVSP; (b) increase significantly in C57BL/6J mice. Serum ANP levels also increase significantly after 3 weeks of chronic hypoxia (c). The association between serum ANP and RV mass is shown for 15 wild‐type C57BL/6J mice in (d). These include normoxic controls (red circles, *n* = 5), 1 week CH‐PH (blue squares, *n* = 5), and 3 week CH‐PH (blue triangles, *n* = 5) animals. *r*_s_ = Spearman's correlation coefficient. In (e), quantitative PCR shows that RV atrial natriuretic peptide (*Nppa*) mRNA is not increased after 3 weeks of CH‐PH, but (f) lung neprolysin 3 receptor (*Npr3*; responsible for ANP catabolism in chronic hypoxia) mRNA is significantly reduced. *p*‐values are for Student's *t*‐test

The murine model of CH‐PH is a well‐characterized model associated with RV hypertrophy and increased RV systolic pressure (RVSP), but not RV failure (Kolb et al., 2015). As anticipated, 3 weeks of CH‐PH significantly increased RVH and RVSP in wild‐type C57BL/6J mice (Table 1 and Figure 1a,b). These changes were associated with a fivefold increase in serum ANP (Figure 1c). Serum ANP levels strongly correlated with RV mass (*r*_S_ =0.67, *p* = 0.008; Figure 1d), but not LV +S mass (*r* = −0.04, *p* = 0.88, not shown). Increased serum ANP was not associated with increased RV *Nppa* transcription (Figure 1e). Rather, increased serum ANP was associated with an 80% reduction in lung Neprolysin‐3 receptor (*Npr3*) expression (Figure 1f). The *Npr3* receptor is highly expressed in lung endothelial cells and lacks an intracellular guanylate cyclase domain; binding of NPs to *Npr3* leads to cellular internalization and lysosomal degradation of ANP (Kolb et al., 2015). Previous studies have demonstrated that increased circulating ANP in the chronic hypoxia model is driven largely by selective downregulation of lung *Npr3* expression (Francis et al., 2011; Sun et al., 2000; Werner et al., 2016).

**TABLE 1:** RV remodeling and hemodynamics in the CH‐PH model

	C57 BL/6J			*Pde9a^+/+^*		*Pde9a^−/−^*		*P* int	*P* Exp	*P* Gen
	Norm	CH	*P* (*t*‐test)	Norm	CH	Norm	CH			
*n*	5	5		10	12	10	10			
BW (g)	30.0 (0.6)	24.0 (1.2)	<0.0001	27.2 (5.2)	24.1 (2.7)	24.9 (2.7)	22.7 (4.6)	0.74	0.04	0.14
RV/LV+S (mg/mg)	0.24 (0.02)	0.34 (0.02)	<0.0001	0.22 (0.03)	0.33 (0.04)	0.24 (0.03)	0.33 (0.05)	0.36	<0.0001	0.54
RV/BW (mg/g)	0.79 (0.04)	1.16 (0.13)	0.0003	0.71 (0.10)	1.16 (0.11)	0.76 (0.06)	1.18 (0.25)	0.8	<0.0001	0.4
LV+S/BW (mg/g)	3.32 (0.13)	3.37 (0.23)	0.64	3.25 (0.27)	3.53 (0.27)	3.21 (0.19)	3.62 (0.50)	0.51	0.0002	0.79
RVSP (mm Hg)	20.2 (3.2)	29.9 (2.4)	0.0006	17.3 (2.1)	27.6 (3.5)	18.3 (1.6)	26.5 (3.7)	0.22	<0.0001	0.98
HR (bpm)	480 (43)	355 (32)	0.0007	508 (39)	424 (47)	490 (39)	428 (55)	0.42	<0.0001	0.6
dP/dt max (mm Hg × s^−1^)	1100 (250)	1178 (109)	0.55	973 (185)	1337 (317)	1036 (161)	1238 (309)	0.31	0.001	0.82
dP/dt min (mm Hg × s^−1^)	−1019 (302)	−1119 (159)	0.53	−846 (179)	−1370 (368)	−932 (134)	−1217 (348)	0.18	<0.0001	0.7

Data are mean and SD for wild‐type C57BL/6J, *Pde9a* ^−/−^ knockout, and *Pde9a* ^+/+^ littermates exposed to normoxia (Norm) or chronic hypoxia (CH) for 3 weeks. *p*_(_ *_t_*_‐test)_ is for comparison of means between normoxic and chronic hypoxia C57BL/6 mice. *p*_Int_ represents the effect of genotype on exposure (Norm vs CH) in *Pde9a* ^+/+^ and *Pde9a* ^−/−^ mice, as measured by two‐way ANOVA. *Post*‐*hoc* comparisons in the transgenic group represented by *p*_Exp_ (Norm vs CH) and *p*_Gen_ (*Pde9a* ^−/−^ vs. *Pde9a* ^+/+^).

Abbreviations: BW, body weight; d*P*/d*t*_max_ = maximal rise in RV pressure during contraction; d*P*/d*t*_min_ = maximal rate of RV pressure decline during relaxation; HR = heart rate; LV +S, left ventricle and septum; RV, right ventricle; RVSP, right ventricular systolic pressure.

### 3.2 Increased circulating ANP is not associated with increased cGMP‐dependent signaling

**FIGURE 2:**
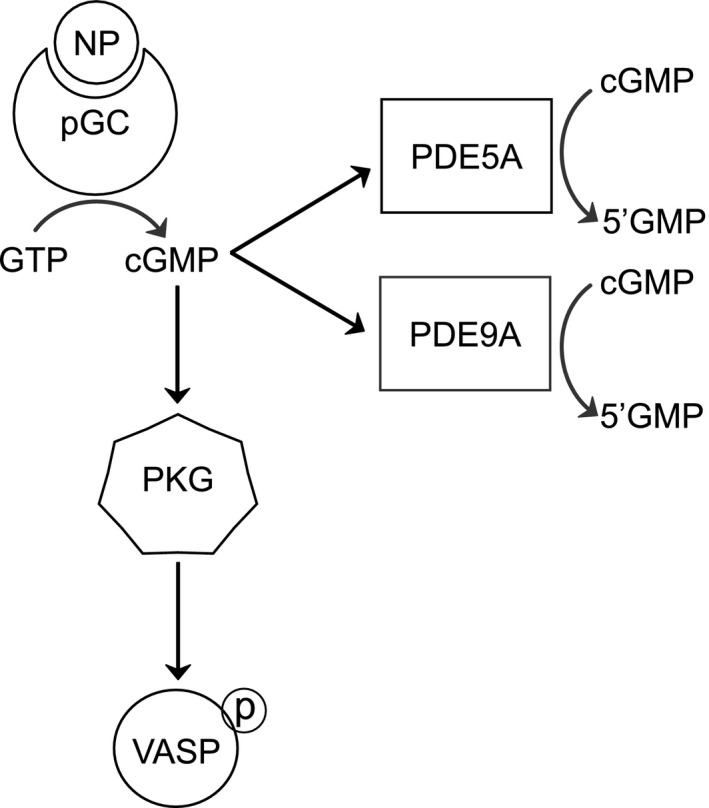
Schematic of signaling pathways examined. Atrial natriuretic peptide (NP) binds membrane‐associated guanylyl cyclase (GC) to catalyze the synthesis of cyclic guanosine 3′,5′‐monophosphate (cGMP) from guanosine 5′‐triphosphate (GTP). cGMP‐dependent protein kinase (PKG) mediates several downstream effects, including phosphorylation and activation of vasodilatory‐stimulated phosphoprotein (VASP). cGMP also activates several phosphodiesterase (PDE) enzymes that can hydrolyze cGMP to guanosine monophosphate (5′GMP), effectively downregulating the signal

**FIGURE 3:**
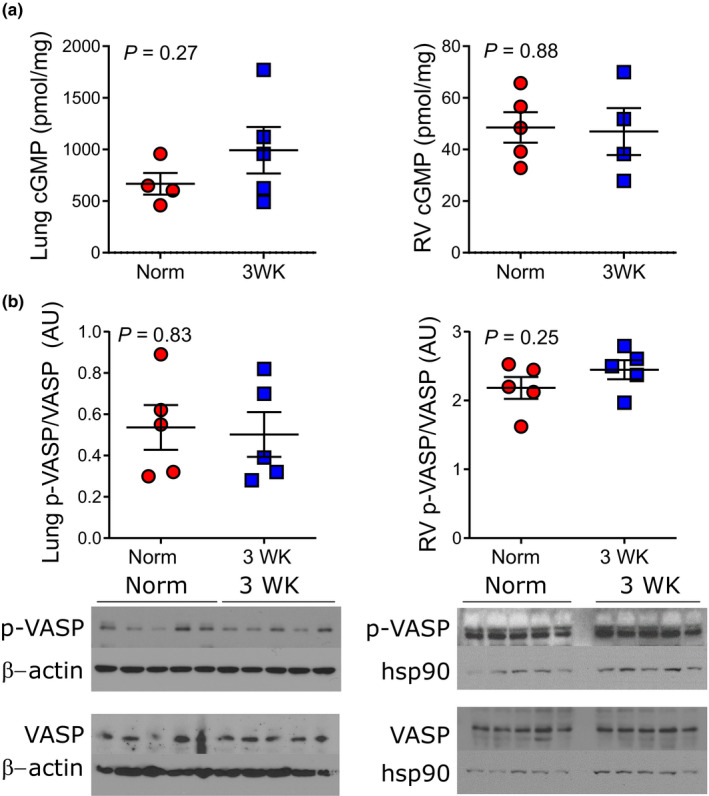
CH‐PH does not increase lung or RV cGMP levels or VASP phosphorylation in C57BL/6J mice. In (a), lung and RV cGMP levels are shown after 3 weeks of CH‐PH in wild‐type C56BL/6J mice. In (b), western blot demonstrates that ser239 phosphorylation of vasodilator‐stimulated phosphoprotein (VASP; surrogate for PKG activity) is not increased in the lung (left) or RV (right) tissue homogenates. *p*‐values are for Student's *t*‐test. AU = arbitrary units

**FIGURE 4:**
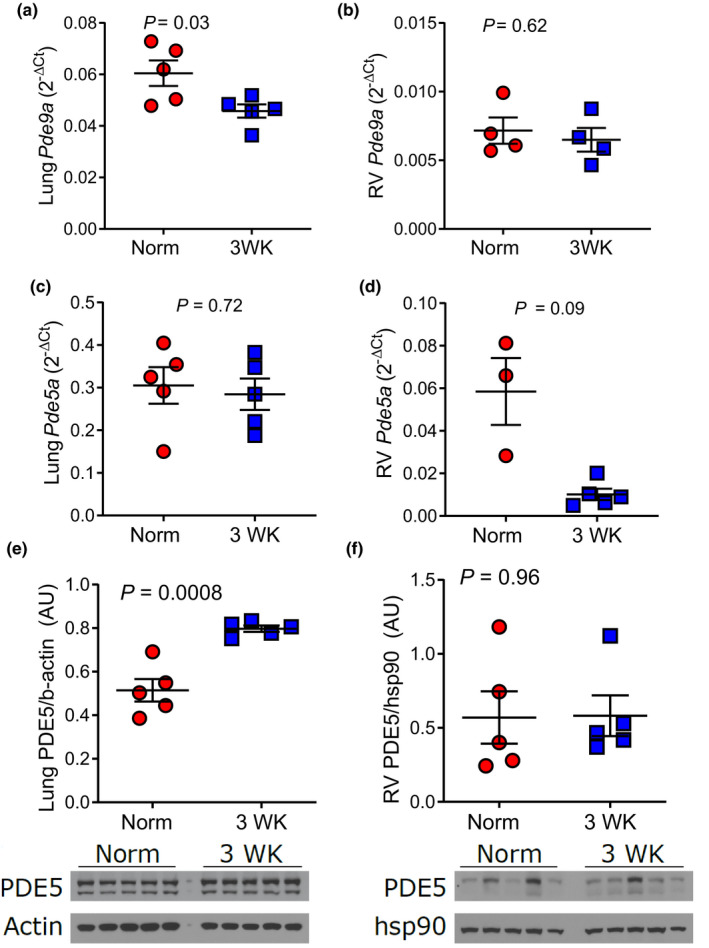
PDE expression in C56BL/6J mice exposed to CH‐PH. After 3 weeks of CH‐PH, levels of *Pde9a* mRNA are reduced in lung (a) and not increased in RV tissue homogenates (b). Similarly, there are no increase in *Pde5a* mRNA in the lung (c) or RV (d) samples after 3 weeks of CH‐PH. In (e), western blot demonstrates a significant increase in PDE5A protein in the lung, but not RV (f) tissue after 3 weeks of CH‐PH. *P*‐values are for Student's *t*‐test. AU = arbitrary units

After establishing an association between CH‐PH and ANP, we measured cGMP levels and activation of cGMP downstream effectors in lung and RV homogenates from C57BL/6J mice exposed to CH‐PH (see Figure 2 for schematic of signaling pathways assessed). Despite observed increases in circulating ANP, CH‐PH did not increase cGMP levels in the lung (993 ± 502 pmol/mg vs 667 ± 210 pmol/mg; *p* = 0.27) or RV (47 ± 18 pmol/mg vs 49 ± 13 pmol/mg; *p* = 0.88) tissue (Figure 3a). Similarly, CH‐PH did not increase phosphorylation of the PKG substrate VASP (vasodilator‐stimulated phosphoprotein) in the lung or RV tissue (Figure 3b). Finally, CH‐PH had minimal impact on cGMP‐dependent PDE expression. In the lung, *Pde9a* transcription decreased after 3 weeks of CH‐PH (Figure 4a), whereas *Pde5a* mRNA was unchanged (Figure 4c). We observed a modest (1.6‐fold) but statistically significant increase in lung PDE5A protein levels after 3 weeks of CH‐PH (*p *= 0.0008; Figure 4e). In the RV, CH‐PH did not increase mRNA levels of *Pde9a* (Figure 4b) or *Pde5a* (Figure 4d) or PDE5A protein (Figure 4f).

### 3.3 PDE9A deficiency is not associated with attenuated lung or RV remodeling in CH‐PH

**FIGURE 5:**
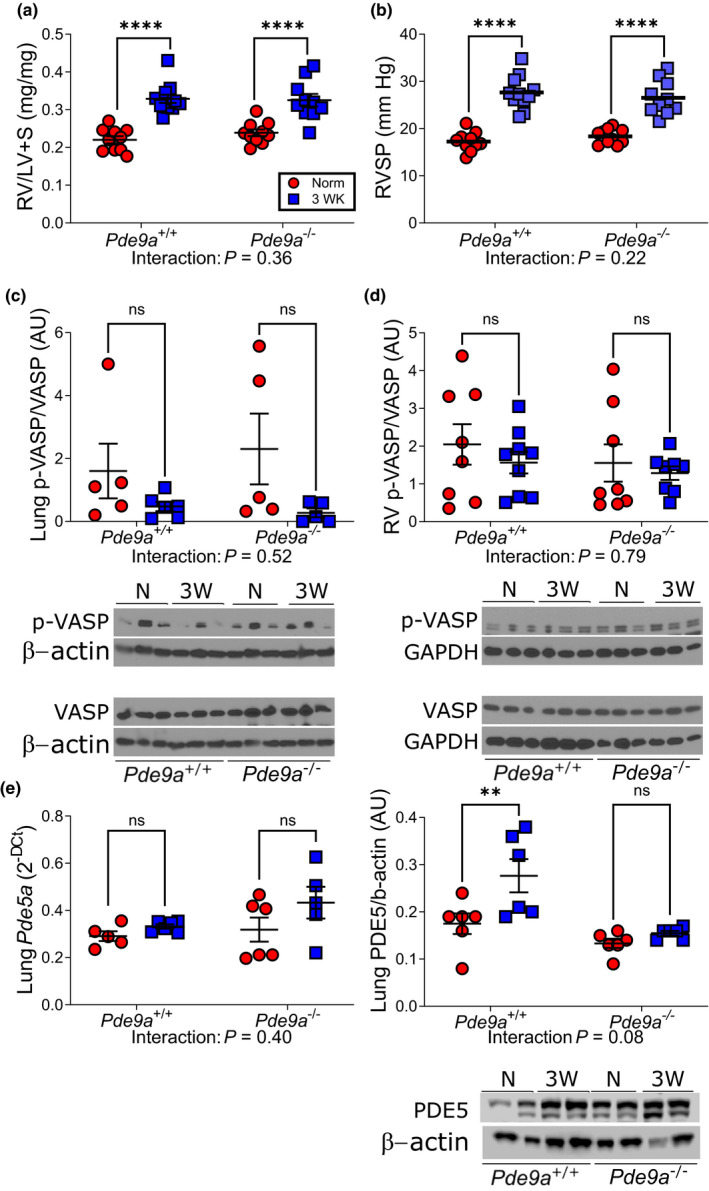
VASP phosphorylation and PDE5A expression in knockout mice. After 3 weeks of exposure to chronic hypoxia, observed increases in RV mass (relative to LV+S = Fulton Index; a) and RV systolic pressure (RVSP; B) do not differ between PDE9A knockout mice (*Pde9a* ^−/−^) and *Pde9a* ^+/+^ littermate controls. *Pde9a* ^−/−^ mice exposed to chronic hypoxia do not have increased lung (c) or RV (d) phosphorylation of the protein kinase G substrate VASP (vasodilator‐stimulated phosphoprotein) compared with *Pde9* ^+/+^ littermates. In lung homogenates (e), there was no significant increase in lung *Pde5a* mRNA in either genotype after 3 weeks of CH‐PH, and no difference in PDE5A protein levels between *Pde9a* ^+/+^ and *Pde9a* ^−/−^ mice. Interaction = *p*‐value for significant interaction by two‐way ANOVA. ****p < 0.0001 in *post*‐*hoc* multiple comparison; ns = not significant in *post*‐*hoc* multiple comparison. N = normoxia; 3WK = chronic hypoxia, 3 weeks

To determine whether PDE9A attenuates protective cGMP signaling and promotes PH development in the murine CH model, we compared RVH, hemodynamics, VASP phosphorylation, and PDE expression between *Pde9a* ^−/−^ mice and *Pde9a* ^+/+^ littermates subjected to 3 weeks of CH‐PH. As shown in Table 1, there were no differences in baseline RV or LV mass between strains. PDE9A deficiency did not attenuate RVH (Figure 5a) or RVSP (Figure 5b) following exposure to CH‐PH. Sex did not alter the relationship between exposure and any hemodynamic variable (Table S1), though there was a trend toward attenuation of CH‐PH‐induced RVSP in *Pde9* ^−/−^ female mice (*p* = 0.05). There was no difference in lung (Figure 5c) or RV (Figure 5d) VASP phosphorylation in *Pde9a* ^−/−^ mice versus *Pde9a* ^+/+^ controls at baseline or after CH‐PH. There was no difference in CH‐PH‐induced PDE5A expression between Pde9a^−/−^ mice and Pde9a^+/+^ littermates (Figure 5e).

## 4 DISCUSSION

In this study, we showed that CH‐PH is associated with increased serum ANP levels but not increased cGMP‐dependent signaling in RV or lung homogenates. More importantly, we show for the first time that CH‐PH is not associated with increased RV or lung expression of *Pde9a* and that PDE9A deficiency is not sufficient to promote cGMP signaling or attenuate the CH‐PH phenotype. Taken together, these findings argue against a prominent role for PDE9A in CH‐PH.

The relationship between NP‐dependent signaling and CH‐PH is complex. Multiple previous studies have used knockout and transgenic mice to demonstrate a protective role for NPs in the CH‐PH model (Chen et al., 2006; Klinger et al., 1993, 1999; Sun et al., 2000; Werner et al., 2016). ANP‐knockout and ANP receptor (guanylyl cyclase‐A; GC‐A)‐knockout mice demonstrated baseline (normoxic) increases in myocardial mass (RV and LV) and pulmonary vessel muscularization and more severe PH after exposure to 3–6 weeks of chronic hypoxia (Chen et al., 2006; Klinger et al., 1999; Sun et al., 2000; Werner et al., 2016). Conversely, transgenic mice overexpressing ANP were protected from pulmonary vascular muscularization, RVH, and RVSP in the CH‐PH model (Klinger et al., 1993). These findings suggest that endogenous ANP prevents spontaneous PH, whereas ANP overexpression protects against CH‐PH. However, protective levels of ANP reported in transgenic mice (10^3^–10^4^ pg/ml) were 10–100‐fold higher than endogenous levels induced in our C57BL/6 mice after exposure to CH‐PH, consistent with a previous report (Sun et al., 2000). Therefore, while it is clear that ANP deficiency exacerbates CH‐PH, it seems likely that supra‐physiologic ANP levels are necessary to attenuate the phenotype. Furthermore, recent evidence showed that chronic hypoxia directly downregulates pulmonary endothelial cell GC‐A expression and activity (Werner et al., 2016), which could further limit the protective effects of the modest ANP upregulation observed in the CH‐PH model.

Potential downstream effectors driving the protective effects of ANP in the CH‐PH model have not been elucidated. In a murine model of chronic LV pressure overload, NP‐dependent cGMP‐PKG signaling attenuated myocardial hypertrophy and reversed LV dysfunction when PDE9A was inhibited (Lee et al., 2015). We did not find definitive evidence of increased cGMP‐PKG signaling in the CH‐PH model with wild‐type or PDE9A‐deficient mice. CH‐PH did not increase levels of RV or lung cGMP, and we observed no increase in phosphorylation of the PKG substrate VASP. We did observe a modest increase in lung PDE5A protein levels in the CH‐PH model, but stimuli other than increased cyclic nucleotide levels (like oxidative stress) are known to increase PDE5A expression (Francis et al., 2011). In a previous report, Sun et al. (2000) showed increased cGMP levels in lung tissue from wild‐type mice exposed to CH‐PH for 5 weeks, though expression and activity of downstream effectors were not reported. This difference may be explained by the longer exposure in the previous study or may be related to differential sensitivity of the detection methods used, as we measured cGMP levels 1000 times higher. Alternative signaling pathways downstream from ANP may play an important role in CH‐PH. In the previous study using endothelial cell GC‐A knockout mice worsened CH‐PH was associated with upregulation of lung angiotensin‐converting enzyme/angiotensin II signaling intermediaries, and losartan reversed the phenotype (Werner et al., 2016). This observation highlights the potential importance of other non‐cGMP‐dependent pathways downstream from ANP in the CH‐PH model.

Despite these limitations, we believe that this study provides an important first step to evaluate the role of PDE9A in an animal PH model. Unfortunately, we were limited in our ability to measure PDE9A protein levels due to the lack of specificity of our anti‐PDE9A antibodies. Nevertheless, *Pde9a* transcription actually declined in lung homogenates and was unchanged in the RV following exposure to CH‐PH. More importantly, PDE9A deficiency did not alter the CH‐PH phenotype in knockout mice. These findings suggest a limited role for PDE9A in CH‐PH. In previous studies demonstrating that *Pde9a* ^−/−^ mice were protected from LV hypertrophy and failure induced by transverse aortic constriction (TAC) (Lee et al., 2015), TAC was associated with more severe ventricular pressure overload, increased expression of ventricular *Nppa* (not observed here), and ample evidence of LV failure (reduced fractional shortening, LV dilation, interstitial fibrosis). In addition, TAC was severe enough to increase *Pde9a* transcription and cGMP levels in the LV. By comparison, we observed a modest increase in ANP using a model we have previously associated with preserved RV function (Kolb et al., 2015). A PH model associated with more severe pulmonary vascular remodeling and RV loading (e.g., rat model of SU5416 and CH or pulmonary artery banding) may be required to sufficiently stimulate important protective NP‐dependent signaling pathways in lung and/or heart and evaluate the role of PDE9A.

A final important consideration for future studies is the potential synergy between PDE9A and PDE5A in regulating cGMP in any PH model. It is possible that both isoforms need to be suppressed to increase cGMP signaling. This hypothesis is supported by a previous study of CH‐PH where rats were treated with ecadotril, a neutral peptidase inhibitor that increases endogenous NP levels (Baliga et al., 2008). Treatment with ecadotril and sildenafil attenuated CH‐PH more effectively than sildenafil alone. In our study, PDE5A protein levels were modestly increased in CH‐PH lungs of wild‐type C57BL/6J mice, but PDE9A deficiency did not alter PDE5A expression (basal or CH‐PH induced). It is possible that an alternative PDE isoform capable of hydrolyzing cGMP was upregulated in the *Pde9a* knockout mice. If PDE9A were the sole or primary PDE responsible for attenuating the effects of NP‐dependent myocardial cGMP signaling, we would hypothesize that *Pde9a* knockout mice would have increased RV and LV mass at baseline, as observed in ANP‐transgenic mouse studies (Klinger et al., 1993). We did not observe any increase in baseline RV or LV mass in our *Pde9a* knockout mice, arguing for the importance of PDE redundancy in these signaling pathways.

## 5 CONCLUSIONS

In sum, PDE9A deficiency did not attenuate CH‐PH in mice. Our findings highlight the complexity of endogenous NP‐dependent signaling in CH‐PH and the limitations of currently available animal models. However, given the considerable potential for the clinical benefit of PDE9A inhibition, either alone or in combination with PDE5A inhibitors, we believe that further studies in more robust PH models are warranted.

## CONFLICT OF INTEREST

The authors have no relevant conflicts to disclose.

## AUTHOR CONTRIBUTIONS

TMK, MD, DAK, and PMH contributed important intellectual content to the conception and design of this study. Data acquisition and analysis were performed by TMK and LJ. The manuscript was drafted by TMK. Critical revisions for important intellectual content were contributed by TMK, MD, LJ, DAK, and PMH. The final version was approved by all authors.

## Supporting information



Figure S1

Table S1
