# Supplementary material for: PDE9A deficiency does not prevent chronic‐hypoxic pulmonary hypertension in mice
Source: Physiol Rep. 2021 Sep 27;9(18):e15057. doi: 10.14814/phy2.15057 (PMC8474007; doi:10.14814/phy2.15057)
Supplement: Supplementary file 2 — Table S1 [file PHY2-9-e15057-s002.docx]

**Supplemental Table 1: RV remodeling and Hemodynamics in Knockout Mice by Sex**

Data are mean and (SD) for *Pde9a^-/-^* and *Pde9a^+/+^* littermates exposed to normoxia (Norm) or chronic hypoxia (CH) for 3 weeks. Analyses are grouped by the sex of animals in each genotype. *P_int_* represents the effect of genotype on exposure (Norm vs. CH) in *Pde9a^+/+^* and *Pde9a^-/-^* mice, as measured by 2-way ANOVA. *Post-hoc* comparisons in the transgenic group represented by *P_Exp_* (Norm vs. CH) and *P_Gen_* (*Pde9a^-/-^* vs. *Pde9a^+/+^*). BW = body weight; RV = right ventricle; LV+S = left ventricle and septum; RVSP = right ventricular systolic pressure; HR = heart rate; dP/dt_max_ = maximal rise in RV pressure during contraction; dP/dt_min_ = maximal rate of RV pressure decline during relaxation.
